# Clinical Simulation Program for the Training of Health Profession Residents in Confidentiality and the Use of Social Networks

**DOI:** 10.3390/nursrep14040221

**Published:** 2024-10-17

**Authors:** Alejandro Martínez-Arce, Alberto Bermejo-Cantarero, Laura Muñoz de Morales-Romero, Víctor Baladrón-González, Natalia Bejarano-Ramírez, Gema Verdugo-Moreno, María Antonia Montero-Gaspar, Francisco Javier Redondo-Calvo

**Affiliations:** 1 Advanced Clinical Simulation Center, General University Hospital of Ciudad Real (HGUCR), 13005 Ciudad Real, Spain; 2 Faculty of Nursing, Universidad de Castilla-La Mancha, 13005 Ciudad Real, Spain; 3 Faculty of Medicine, Universidad de Castilla-La Mancha, 13005 Ciudad Real, Spain

**Keywords:** high-fidelity simulation training, confidentiality, privacy, health education, nursing education research, professionalism, social media

## Abstract

Background: In the transition to a professional learning environment, healthcare professionals in their first year of specialized postgraduate clinical training (known as residents in Spain) are suddenly required to handle confidential information with little or no prior training in the safe and appropriate use of digital media with respect to confidentiality issues. The aims of this study were: (1) to explore the usefulness of an advanced clinical simulation program for educating residents from different healthcare disciplines about confidentiality and the dissemination of clinical data or patient images; (2) to explore the use of social networks in healthcare settings; and (3) to explore participants’ knowledge and attitudes on current regulations regarding confidentiality, image dissemination, and the use of social networks; Methods: This was a cross-sectional study. Data were collected from all 49 first-year residents of different health professions at a Spanish hospital between June and August 2022. High-fidelity clinical simulation sessions designed to address confidentiality and health information dissemination issues in hospital settings, including the use of social networks, were developed and implemented. Data were assessed using a 12-item ad hoc questionnaire on confidentiality and the use of social media in the healthcare setting. Descriptive of general data and chi-square test or Fisher’s exact test were performed using the SPSS 25.0 software; Results: All the participants reported using the messaging application WhatsApp regularly during their working day. A total of 20.4% of the participants stated that they had taken photos of clinical data (radiographs, analyses, etc.) without permission, with 40.8% claiming that they were unaware of the legal consequences of improper access to clinical records. After the course, the participants reported intending to modify their behavior when sharing patient data without their consent and with respect to how patients are informed; Conclusions: The use of advanced simulation in the training of interprofessional teams of residents is as an effective tool for initiating attitudinal change and increasing knowledge related to patient privacy and confidentiality. Further follow-up studies are needed to see how these attitudes are incorporated into clinical practice.

## 1. Introduction

Privacy is recognized as a fundamental right of the individual to honor and freedom [1,2]. Everything that belongs to the sphere of privacy is confidential. The right to confidentiality protects the use of intimate information [3]. It is considered an absolute ethical principle in the healthcare field and one of the central concepts of professionalism [4,5].

In the digital era, the confidentiality of health information has become an issue of growing concern [6]. Technological development and the evolution towards a digitized and connected clinical history model, added to the possibilities of exchanging medical information brought about by the rise of social networks, facilitate the dissemination of confidential clinical data, infringing patients’ right to privacy, increasing the risk of legal and disciplinary problems, and eroding user confidence by providing poor quality information [7]. Thus, key regulations, such as General Data Protection Regulation (GDPR) [8] in Europe, or the Spanish Personal Data Protection Law and guarantee of digital rights [9], along with professional codes of ethics, emphasize the need to protect patient information. These frameworks outline strict guidelines for handling and sharing confidential data, especially in digital and social media contexts, to prevent breaches of privacy.

Due to their mass use and relationship to confidentiality, social networks will have implications for health sciences education. However, to date, little content has been included in undergraduate curricula to help students use digital media safely and appropriately [10,11,12].

In the transition from a purely academic to a professional learning environment, resident health professionals are suddenly faced with moral judgments about the disclosure of clinical information. This shift is complicated and poses a risk to the privacy of patient information [13,14]. The moral dilemmas that they face are complex and multidimensional. On the one hand, they must learn to prioritize and apply moral values in the context of real-world clinical interactions. On the other hand, there is a conflict between internal motivations and external circumstances that constrain decision making in a hierarchical and multiprofessional environment [15].

In this context, experts advise addressing, in postgraduate training programs, specific hypothetical situations that pose the risk of an avoidable breach of confidentiality [16,17,18,19]. In this line, clinical simulation has been postulated as an effective learning tool. Clinical simulation is based on the experiential learning model and constructivist theories [20,21]. Its integration into the specialized training curriculum is recommended, in order for future specialists to be exposed to previously designed simulated experiences that are similar to real-life ones in a safe environment before they are required to manage such situations in actual clinical practice [22,23]. This improves individuals’ safety and confidence and promotes therapeutic communication skills in students [24,25,26,27]. In addition, it makes it possible for professionals from different disciplines to be trained together.

The potential of advanced clinical simulation to improve empathy with patients [28] and raise awareness of privacy [29] and humanization [30] is substantial. However, to the best of our knowledge, its usefulness in training residents from different healthcare disciplines in aspects related to confidentiality and the use of social networks has not been previously explored.

The objectives of our study were: (1) to explore the usefulness of training residents from different healthcare disciplines using an advanced clinical simulation program on confidentiality and the dissemination of clinical data or patient images; (2) to explore the use of social networks in healthcare settings; and (3) to explore the participants’ knowledge and attitudes on current regulations on confidentiality, image dissemination, and the use of social networks.

## 2. Materials and Methods

### 2.1. Study Design and Sample

This cross-sectional study describes the development and implementation of high-fidelity clinical simulation sessions designed to address issues of confidentiality and the dissemination of health information in the hospital setting, including the use of social networks, image sharing, and current regulations on confidentiality and the disclosure of clinical data. Participants’ attitudes and knowledge, and the effectiveness of the sessions, were assessed using a cross-sectional analysis of responses to a self-administered questionnaire completed at the end of each simulation course. Data were collected in June and August 2022. First-year residents from different health professions were included: Nursing Internal Residents (NIRs), Pharmacy Internal Residents (PhIRs), Medicine Internal Residents (MIRs), and Psychology Internal Residents (PIRs). The training was carried out at the Advanced Simulation Center of the General University Hospital of Ciudad Real (Spain). Participation in these training courses was mandatory for all the residents during their first weeks of incorporation into the activity of the hospital and the accredited primary care centers. Health professionals in the second and subsequent years of specialized formation, as well as other professionals without specialized postgraduate training, were excluded from the study. A convenience sample of 49 participants (all the potential participants) was obtained from eight clinical simulation sessions.

### 2.2. Procedure and Study Variables

For this study, five simulation sessions were conducted. Each training session was designed to last 6 h with a maximum of 10 participants per session. Each of these workshops was based on the resolution of three clinical cases in a realistic simulation environment (three cases per 6-h session). The theoretical framework on confidentiality and the use of social networks that guided the definition of the scenarios and the objectives of the simulations were as follows: first, we based our approach on the principles of medical ethics, particularly the duty of confidentiality, as outlined in Beauchamp and Childress’ Four Principles of Biomedical Ethics [31]. These principles—autonomy, beneficence, non-maleficence, and justice—provided the ethical foundation for discussing privacy and appropriate boundaries in the handling of patient information. In addition, we integrated concepts of digital professionalism, as described by Ellaway et al. [10], that emphasize maintaining professional standards in digital spaces, including social networks. This framework addresses how healthcare professionals should navigate online environments while respecting patient confidentiality, institutional policies, and personal integrity. In addition, we considered the context of current privacy legislation to ensure that the scenarios reflected real-world legal considerations regarding patient data and misuse.

The instructors used the Laerdal SimMan high-fidelity patient simulator, supported by the Laerdal Learning Application (LLEAP) simulation software version 7.3.0 (Laerdal Medical Corporation, Wappingers Falls, NY, USA). This high-fidelity clinical simulation mannequin is an advanced training tool designed to realistically mimic human physiology and responses in medical scenarios. It can simulate a wide range of clinical conditions, including vital signs, breathing, heart rhythms, and even verbal communication. When necessary, a member of the simulation teaching staff with acting experience performed the role of the standardized patient or the inexperienced professional introducing the conflict to be resolved. The participants in the different editions, who had not received any prior information or material relating to the cases, faced the same scenarios and were taught by the same teaching team to guarantee the homogeneity of the training received after agreeing not to transmit information about the course to the following groups of participants (by signing a confidentiality agreement). A work team, made up of clinical simulation instructors certified by Harvard´s Center for Medical Simulation (nurses and physicians) and those responsible for managing the teaching of the residents’ activities, designed the simulated clinical scenarios based on the previously agreed training objectives related to confidentiality and the dissemination of health information. The simulation scenarios were designed according to the guidelines of the Center for Medical Simulation (Harvard University, Boston, MA, USA).

Each of the five training sessions with the clinical simulation were organized according to the following structure:

- Information about the study, signing the informed consent form, and signing the confidentiality document (15 min).
- Introduction of participants and instructors. Motivations for attending the course, previous experience in clinical simulation, and expectations of the training they will receive were discussed individually (30 min).
- Prebriefing or introduction to the simulation (30 min): Before starting the scenarios, a brief introduction was given to inform the participants of the rules of the clinical simulation and the development of the session.
- Demonstration of the workplace and simulator. Explanation of the functions and limitations of the high-technology patient simulator (30 min).
- Briefing: Scenario presentation (10 min per case). The course instructor assigned an interprofessional team of five persons to each scenario and provided the participants with the information necessary for the correct development of the case. Each resident participated in at least one of the simulations.
- Action: Development of the clinical case by simulation (15 min). In each 6-h session, three scenarios were simulated, each lasting 15 min. The theoretical framework that guided the organization of the topics in the simulation scenarios was based on experiential learning and constructivist theory. The scenarios were based on the simulation of different clinical situations in which conflicts were generated related to decision making in the area of confidentiality, information provided to patients and family members, and the dissemination of clinical information, with a special emphasis on the use of social networks (Table 1). The scenario was observed live, in an adjacent room, by the instructors and the rest of the participants, and each scenario was recorded for later analysis, with the prior consent of the participants.
- Debriefing: All participants, including observers, took part in a group debriefing led by the instructor immediately after each scenario. Each simulated clinical case, with its subsequent debriefing, lasted approximately 60 min. In the debriefing, key points related to the previously agreed teaching objectives were identified and analyzed: management of confidential data, maintenance of personal privacy, regulations on access to medical records, legislation on the dissemination and use of patient images, and assessment of the appropriate environment in which health system users and their families are informed.
  The simulated clinical cases were analyzed using the technique of debriefing with good judgment [32,33].
- Completion of the questionnaire after the course (15 min).

### 2.3. Instrument

Knowledge and attitudes on aspects related to confidentiality and the dissemination of health information were assessed using an ad hoc questionnaire based on the previous literature [34,35]. Following the development of the questionnaire by the principal researcher, a panel of experts checked the questions included. The experts evaluated the clarity and difficulty level of the questions, the accuracy of the correct answer, and the time usage. The scale comprised 12 items. This questionnaire included two multiple-response items related to the use of social networks; four items on taking, receiving, and disseminating photographs of patients and/or their clinical data without permission; two items related to knowledge of the legislation concerning access to medical records and the use of clinical data for scientific purposes; and four items on behavior modification after the training session with the clinical simulation.

Data on sociodemographic variables were collected, including age, sex, and field of specialized training (NIR, PhIR, MIR, and PIR). All the participants completed the questionnaires within minutes of completing the simulation exercises.

### 2.4. Data Analysis

Descriptive statistics were used to calculate frequencies and percentages. Age is presented as mean and standard deviation, by sex. The multiple-response questions were dichotomized (YES/NO) for analysis. Associations between qualitative variables were examined using the chi-square test or Fisher’s exact test. Statistical significance was set at *p* < 0.05. Data were analyzed using the SPSS program, version 25.0 (IBM, Armonk, NY, USA).

### 2.5. Ethical Approval and Informed Consent

Written informed consent was obtained for participation. In addition, participants signed a non-disclosure agreement and consented to the recording of the simulation scenarios. This study was exempt from institutional review board approval because it did not involve personally identifiable information. This study was conducted in accordance with the tenets of the Declaration of Helsinki. Participants were free to refuse to participate or to withdraw from the study at any time.

## 3. Results

Table 2 shows the descriptive characteristics of the sample. A total of 49 questionnaires were analyzed. Of the participants, 13 were men (29.53%) and 36 were women (73.47%). The mean age was 26.45 years (SD = 3.35), with no differences between men (26.92, SD = 4.94) and women (26.27, SD = 2.63) (*p* = 0.146). Most of the residents belonged to the disciplines of medicine (63.3%) and nursing (26.5%). All the participants reported regular use of the WhatsApp messaging application v. 2.22 (Meta Platforms Inc., Mountain View, California, USA) both during and outside of their working day. Although most of them stated that they used Instagram (83.7%), the number of residents using it during work was considerably smaller (34.7%). The next most used social networks overall were Twitter (38.8%) and Facebook (32.7%), although the use of these networks was immensely lower during working hours (8.2% and 4.1%, respectively).

Table 3 shows the frequency and percentage of responses to the different items in the questionnaire. A total of 20.4% of the participants reported having used their cell phones to take photos of clinical data (radiographs, analyses, etc.) without permission during the first weeks of work (2 NIR, 8 MIR, *p* = 0.564). Four participants confirmed having uploaded photos of either clinical data (4.1%) or of other professionals (2%, and 1%, respectively) to WhatsApp or Telegram or other social networks. When asked whether they had received images of clinical or patient data from co-workers in the recent weeks, 24.9% answered affirmatively, for both clinical data (20.4%) and patient photos (4.1%).

Regarding access to the clinical records of patients, family members, or colleagues who were not being treated directly by them in a healthcare process, and without having obtained their consent, 40.8% of the residents responded that they were unaware of the legal consequences of improper access to clinical records and that they thought that they could access them freely because they were healthcare professionals. Likewise, 26.5% acknowledged that they were ignorant of the obligation to request specific consent to publish, for scientific purposes, an image or clinical data of a patient. No differences were found regarding sex or specialty in terms of knowledge of the regulations on access to medical records (*p* = 0.591) or knowledge of the need for consent to publish images for scientific purposes (*p* = 0.430). Ignorance of the prohibition of access to the medical records of patients who are not directly under their care was significantly related to taking photos of clinical data with personal cell phones (*p* = 0.035), although it was not related to their dissemination via WhatsApp or Telegram (*p* = 0.110) or other social networks (*p* = 0.089), or to the ignorance of the need for consent for the dissemination of images for scientific purposes (*p* = 0.274).

A large percentage of the participants (73.5%) confirmed that prior to the course, in their first weeks of joining the hospital, they had witnessed patients being informed in places that were neither appropriate nor specifically set up for this purpose. There were significant differences by specialty (*p* = 0.001), with medical residents having observed the most cases.

After the course, the participants claimed that they intended to modify their behavior when sharing patient data without consent (85.7%) and with respect to the way of informing patients and the management of the environment or context surrounding this information exchange (93.9%). A total of 20.4% of the participants admitted having deleted, after the course, a photo from their personal cell phone or a photo that had been published on a social network. No significant differences were observed between the different healthcare specialties (*p* = 0.896 and *p* = 0.334, respectively). The intention to modify behavior after the course was not related to photo deletion in any of the specialties (*p* = 0.148).

## 4. Discussion

The results of our study show that advanced clinical simulation could help to generate a change of attitude in health professionals in training regarding the dissemination of images through social networks, the way and place in which patients are informed, and the request for consent for the use of images for scientific purposes. It was observed that a considerable percentage of residents use social networks to share or disseminate clinical information through personal devices, especially messaging, which infringes patients’ privacy. The participants in our study showed a lack of training that led to ignorance of the regulations related to the confidentiality of clinical data, access to medical records, or the dissemination of images.

Corroborating the findings of previous studies [16], our data indicate the need for training that specifically addresses situations of breaches of confidentiality that most professionals do not recognize as such. Our clinical simulation training program for residents could be positioned as an effective tool for changing attitudes towards sharing personal data without consent, with some participants even acknowledging the voluntary deletion of photos after the course. The residents were also made aware of the importance of the place in which to inform patients and their families. The methodology used in this study, which encompasses the assessment of objective knowledge, professional ability, and behavioral responses to develop non-technical skills that enhance professionalism, has been shown to be a more effective tool than traditional methods [36]. This is because it provides sufficiently clear and concrete specifications, after confronting them with real ethical dilemmas, on the information that they can publish, such that they can avoid being sanctioned arbitrarily or unpredictably by the administration [37].

As suggested in a previous study [38], the new generations of students in the healthcare field have integrated the use of digital media and social networks into all their life habits, and although this fact in itself does not necessarily imply a breach of confidentiality, it does facilitate the easy and quick exchange of images and information between professionals. This circumstance, moreover, increases the risk of mixing their personal and professional lives, creating profiles in which patients and family members can access images or data on clinical cases [39]. In a 2015 study [40], most medical school representatives reported incidents involving students posting unprofessional content on the Internet. Some of these incidents involved a breach of patient confidentiality (blogs that describe clinical experiences in enough detail to identify patients, or posting patient information on Facebook).

Our findings confirm that most residents, especially medical residents, have witnessed patients being informed in places that, because of their structure and location, were inappropriate for this purpose (i.e., hallways, common areas, shared rooms, or waiting rooms where other people could overhear this information). Coinciding with our results, previous research [41,42,43] has concluded that breaches of confidentiality related to how and where users and families receive information are more common than expected by all members of the patient care team. This situation, to a large extent, is favored by the physical structure of healthcare centers, the proximity of work areas in relation to waiting areas and patient rooms, as well as the unsuitable locations of information areas. Likewise, a considerable number of professionals discuss confidential matters about health system users who can be identified with family and friends or do not take sufficient precautions when talking about patients in public places [16].

Several studies [44,45,46] have highlighted healthcare professionals’ concerns about the confidentiality of the data included in the medical record, mainly those related to inadequate training and improper access by unauthorized users. In our study, 40.8% of residents were unaware that access to the medical record is limited to professionals directly involved in patient care, nor that improper access can have legal consequences. These results are consistent with previous research in which most of the students acknowledged using other professionals’ passwords to access clinical histories and accessing personal data that were unrelated to the current clinical process [47]. Furthermore, although most professionals know when it is legitimate to access a patient’s data, they recognize that they have acted contrary to the norm [34]. This lack of knowledge of current confidentiality regulations could be trained through clinical simulation, as advanced simulation training in health profession education has been consistently associated with significant benefits in knowledge, skill, and behavioral outcomes [48,49].

## 5. Limitations

The present study has several limitations. First, it was limited by the small sample size. Due to the infrastructure and specialized personnel required to carry out the courses, we were unable to expand the number of training places offered. Even so, the sample comprised all the first-year residents in training at our hospital. Second, due to its cross-sectional design, we cannot establish whether the observed intentions to change attitudes are consolidated in the daily practice of the professionals as a real change in the long term. In the future, studies involving different simulation centers and with repeated measurements throughout the course of specialized training could confirm whether these attitudes towards privacy and confidentiality are maintained over time. Furthermore, the measure of knowledge on regulation at the end of a training could be biased by the stimulus received during the training. In future studies, the questionnaire should be administered before and after the simulation. Third, although our results are in line with the existing literature, the questionnaire designed for this study by the research team may not have sufficient capacity to measure the construct for which it was designed. And fourth, participants may have underreported their inappropriate attitudes.

## 6. Conclusions

Our results suggest that training interprofessional teams of residents using an advanced simulation could serve as an effective tool for modifying attitudes and increasing knowledge related to patient privacy and confidentiality. This clinical simulation-based approach is of particular importance in a context in which the use of social networks is massively present in both the professional and personal settings, as it allows residents to face scenarios that recreate the risks of disseminating confidential information in a controlled environment. Although a significant percentage of professionals in training exhibit compliant behaviors, there are still important gaps in confidentiality resulting from the transfer of images without permission through personal devices, how and where patients are informed, and improper access to medical records. Residents should be aware of their professional responsibility when using social networks and be vigilant about the information they share and how they share it. For their part, healthcare centers should ensure the training of their future professionals and develop clear policies on the use of social networks and access to medical records. In this regard, we recommend the integration of simulation training, which is based on the analysis of clinical cases proposing problems related to confidentiality that arise in clinical practice, as a measure to be adopted by healthcare centers in the training curriculum for their residents.

## Figures and Tables

**Table 1:** Description of simulated clinical scenarios.

Scenario(10 min)	Objectives	Brief Description	Staff/Equipment	Debriefing Key Points(45–50 min)
Communication failure	Medical record information management Communication between professionals	In the presence of an error in the administration of medication, one of the professionals seeks to hide it from the patient, the rest of the team, and the patient’s relatives.	High-fidelity mannequin SimMan Laerdal Actor (student trainee with little experience)	Patient identification Confidential data management and truth in communication Importance of the patient as the primary receptor of the information Security error notifications
Non-confidential diagnostic	Transmission of clinical information Assessment of the environment	Two patients, acquaintances from the same town, share a room. Sensitive information about an unwanted pregnancy is transmitted to one of them in the presence of the other.	High-fidelity mannequin SimMan Laerdal Actor (standardized patient)	Maintaining patients’ privacy Assessment of the appropriate environment for providing clinical information to patients and families
Breach of confidentiality in a critical situation	Knowledge of the proper handling of patient images or clinical data Organization of teams in critical situations	Patient with abdominal pain progressing to cardiorespiratory arrest. A professional from the team recognizes the patient as their neighbor and phones his wife.	High-fidelity mannequin SimMan Laerdal Actor (assistant professional)	Regulations on taking and disseminating images of patients Use of social networks, e-mail, and personal telephones for transmitting clinical information Group coordination and teamwork

**Table 2:** Descriptive characteristics of the sample.

	Total (n = 49)	Men (n = 13)	Women (n = 36)	*p*
Specialty, n (%)				0.719
NIR	13 (26.5)	3 (23.1)	10 (27.8)	
PhIR	4 (8.2)	2 (15.4)	2 (5.6)	
MIR	31 (63.3)	8 (61.5)	23 (63.9)	
PIR	1 (2)	0	1 (2.8)	
Social network use, n (%)				
WhatsApp	49 (100)	13 (100)	36 (100)	
Instagram	41 (83.7)	11 (84.6)	30 (83.3)	0.915
Facebook	16 (32.7)	3 (23.1)	13 (36.1)	0.390
Telegram	11 (22.4)	5 (38.5)	6 (16.7)	0.106
Twitter	19 (38.8)	6 (46.2)	13 (36.1)	0.524
TikTok	7 (14.3)	2 (15.4)	5 (13.9)	0.895
Use of social networks in the workplace, n (%)				
WhatsApp	49 (100)	13 (100)	36 (100)	
Instagram	17 (34.7)	4 (30.8)	13 (36.1)	1.000
Facebook	2 (4.1)	0	2 (5.6)	1.000
Telegram	2 (4.1)	0	2 (5.6)	1.000
Twitter	4 (8.2)	2 (15.4)	2 (5.6)	0.284
TikTok	1 (2.0)	0	1 (2.8)	1.000

Data are presented as frequencies and percentages; n (%), for Specialty, Social network use, and Use of social networks in the workplace. NIR: Nurse Intern Resident; PhIR: Pharmacy Internal Resident; MIR: Medicine Internal Resident; PIR: Psychology Internal Resident.

**Table 3:** Distribution of the percentage and frequency of responses to the items in the questionnaire on confidentiality and dissemination of health information.

	Total (n = 49)	Men (n = 13)	Women (n = 36)	*p*
Taking photos of clinical data using personal cell phones; n (%)				0.706
Total	10 (20.4)	3 (23.1)	7 (19.4)	
NIR	2	2 (66.7)	0	
PhIR	0	0	0	
MIR	8 (25.8)	1 (12.5)	7 (30.4)	
PIR	0	0	0	
Photos shared via WhatsApp or Telegram; n (%)				0.334
No	7 (14.3)	1 (7.7)	6 (16.7)	
Clinical data	2 (4.1)	1 (7.7)	1 (2.8)	
Other professionals	1 (2.0)	1 (7.7)	0	
Photos shared via other social networks; n (%)				0.331
No	9 (18.4)	2 (15.4)	7 (19.4)	
Other professionals	1 (2.0)	1 (7.7)	0	
Reception of images taken by colleagues; n (%)				0.694
No	37 (75.5)	10 (76.9)	27 (75.0)	
Clinical data	10 (20.4)	2 (15.4)	8 (22.2)	
Patients	2 (4.1)	1 (7.7)	1 (2.8)	
Knowledge of regulations on access to medical records; n (%)				0.840
Yes	29 (59.2)	8 (61.5)	21 (58.3)	
No	20 (40.8)	5 (38.5)	15 (41.7)	
Knowledge of confidentiality and consent to scientific publication of photos; n (%)				0.288
Yes	36 (73.5)	11 (84.6)	25 (69.4)	
No	13 (26.5)	2 (15.4)	11 (30.6)	
Data sharing behavior modification; n (%)				0.363
Yes	42 (85.7)	10 (76.9)	32 (88.9)	
No	7 (14.3)	3 (23.1)	4 (11.1)	
Witnessing of information being given to patients in an unsuitable environment; n (%)				0.686
Yes	36 (73.5)	9 (69.2)	27 (75.0)	
No	13 (26.5)	4 (30.8)	9 (25.0)	
Behavior modification regarding the environment in which the patients are informed; n (%)				0.168
Yes	6 (93.9)	11 (84.6)	36 (97.2)	
No	3 (6.1)	2 (15.4)	1 (2.8)	
Deletion of photos after the course; n (%)				0.781
Yes	10 (20.4)	3 (23.1)	7 (19.4)	
No	39 (79.6)	7 (76.9)	29 (80.6)	

Data are presented as frequencies and percentages; n (%). NIR: Nurse Intern Resident; PhIR: Pharmacy Internal Resident; MIR: Medicine Internal Resident; PIR: Psychology Internal Resident. *p*-value: differences by gender.

## Data Availability

The data that support the findings of this study are available on request from the corresponding author. The data are not publicly available due to privacy or ethical restrictions.

## Public Involvement Statement

There was no public involvement in any aspect of this research.

## Guidelines and Standards Statement

This manuscript was drafted against the reporting guidelines for observational studies according to the STROBE (Reporting of Observational Studies in Epidemiology) statement.
